# The effect of universal infant vaccination on the prevalence of hepatitis B immunity in adult solid organ transplant candidates

**DOI:** 10.1111/jvh.13414

**Published:** 2020-10-14

**Authors:** Özgür M. Koc, Dirk Kuypers, Lieven J Dupont, Robin Vos, Jan M. Van Keer, Johan Van Cleemput, Geert Robaeys, Astrid Oude Lashof, Matthijs Kramer, Geert Verleden, Jef Verbeek, Frederik Nevens

**Affiliations:** ^1^ Department of Gastroenterology and Hepatology Ziekenhuis Oost‐Limburg Genk Belgium; ^2^ Faculty of Medicine and Life Sciences Hasselt University Hasselt Belgium; ^3^ Department of Medical Microbiology Maastricht University Medical Centre Maastricht the Netherlands; ^4^ School of Nutrition and Translational Research in Metabolism (NUTRIM) University Maastricht Maastricht the Netherlands; ^5^ Department of Nephrology and Renal Transplantation University Hospitals Leuven Leuven Belgium; ^6^ Department of Microbiology Immunology & Transplantation KU Leuven Leuven Belgium; ^7^ Department of Respiratory Diseases University Hospitals Leuven Leuven Belgium; ^8^ Department of Cardiology University Hospitals Leuven Leuven Belgium; ^9^ Department of Gastroenterology and Hepatology University Hospitals Leuven Leuven Belgium; ^10^ Department of Internal Medicine Division of Gastroenterology and Hepatology Maastricht University Medical Centre Maastricht the Netherlands

**Keywords:** hepatitis B, immunity, solid organ transplant, vaccination

## Abstract

**Background:**

Hepatitis B virus (HBV) immunity is recommended to optimize outcomes after solid organ transplantation (SOT). This study assessed the prevalence and predictors of HBV immunity at the time patients were placed on transplant waiting list over a period from 1997 to 2019 in a low HBV endemic region.

**Methods:**

Data were obtained from the University Hospitals Leuven transplant database. Minors and patients with past/current HBV infection were excluded. From 1986, Belgian patients are covered by the universal infant vaccination; therefore, birth cohort was stratified in those born ≥1986 vs <1986.

**Results:**

The study population consisted of 3297 SOT candidates. HBV immunity rate was superior in renal transplant candidates (55.3%), and this number was 21.5%, 15.4% and 16.8% for liver, cardiac and pulmonary transplant candidates, respectively, *P* < .001. Among liver transplant candidates, HBV immunity rate was 14.8% in decompensated cirrhotic patients and 27.9% in those without advanced cirrhosis (*P* < .001). The overall immunity rate increased from 19.3% in period 1997‐2008 to 32.8% in 2009‐2019, *P* < .001. In multivariable analyses, younger age (odds ratio (OR) 95% confidence interval (CI): 0.97‐0.98, *P* < .001) and birth cohort ≥ 1986 (OR 95% CI: 1.18‐2.66, *P* = .006) were associated with increased HBV immunity.

**Conclusion:**

An increase in HBV immunity was observed over a 20‐year period related to the introduction of universal infant HBV vaccination. Nevertheless, this study highlights the low overall HBV immunity at the time of listing for organ transplantation and points out the need of an increased awareness and vaccination strategy at an early disease stage.

**Abbreviations**
BMI: body mass index
CI: confidence interval
HBsAg: hepatitis B surface antigen
HBV: hepatitis B virus
HCV: hepatitis C virus
HLA: human leucocyte antigen
OR: odds ratio
SOT: solid organ transplant

## 1 INTRODUCTION

Hepatitis B virus (HBV) screening of solid organ transplant (SOT) candidates is recommended to optimize outcomes after transplantation.^1^, ^2^ Since the achievement of adequate hepatitis B antibody (anti‐HBs) levels (≥10 mIU/mL) prevents de novo HBV infection acquired during or after transplantation, nonimmune SOT candidates should receive the HBV vaccine series.^3^, ^4^, ^5^

Current licensed HBV vaccines are produced in *Saccharomyces cerevisiae* and vary in the amount of hepatitis B surface antigen (HBsAg) (20, 40 µg).^6^ Upon administration of a standard 3‐dose or 4‐dose vaccine series, >90% of the general adult population will develop an adequate immune response (anti‐HBs level ≥ 10 mIU/mL measured at 1‐3 months after completion of the HBV vaccine series).^7^, ^8^, ^9^, ^10^ Risk factors for a suboptimal immune response after HBV immunization include age ≥ 40 years, male sex, smoking, obesity, immunodeficiency and genetic factors (eg HLA DQ2, DR3 and DR7).^7^, ^8^, ^9^, ^10^, ^11^

It is well‐known that HBV vaccines are less effective in SOT candidates.^12^, ^13^, ^14^, ^15^, ^16^, ^17^, ^18^, ^19^ In cirrhotic patients waiting for liver transplantation, only 10%‐36% develop protective antibody levels after a standard vaccination schedule with 3 or 4 doses of 20 µg HBsAg.^12^, ^20^, ^21^, ^22^ Providing a double‐dose (40 µg) schedule increases the seroprotection rate to 26%‐68%.^14^, ^20^, ^23^, ^24^ A recent systematic review also reported a poorer immune response in patients with end‐stage renal disease with 24%‐42% seroprotection after standard‐dose (20 µg) HBV vaccination and 51%‐69% after double‐dose (40 µg) vaccination.^25^ Low success rates of HBV vaccination have also been reported in cardiac and pulmonary transplant candidates.^26^, ^27^

Recent data on the immunization rate among SOT candidates in a low HBV endemic region and the effect of universal infant vaccination have not been explored thus far.

This study provides information on the prevalence of HBV immunity at the time SOT candidates were placed on the transplant waiting list over the period 1997‐2019 in Belgium, a low endemic region for HBV. The current study also assessed the impact of universal HBV vaccination on HBV immunity at the time of transplant listing. In Belgium, universal infant HBV vaccination began in 1999 with catch‐up vaccination for 10‐ to 13‐year‐olds (birth cohort born in 1986 or after).^28^, ^29^

## 2 PATIENTS AND METHODS

### 2.1 Study design

We conducted a retrospective study at the University Hospitals Leuven, an academic hospital in Leuven with a capacity of 1995 beds. The hospital is involved in transplant care with active programmes of liver, kidney, heart, lung, pancreas, bowel, bone marrow and composite tissue grafts.

Solid organ transplantation candidates between 1 January 1997 to 21 June 2019 were identified from the hospital's transplant database. All included patients had information on HBV serology. Minors (aged < 18 years), patients with current HBV infection (HBsAg positivity) and patients with past HBV infection (HBsAg negative, anti‐HBc positive) were excluded.

Data on transplant diagnosis, age, sex, race, body mass index (BMI), human leucocyte antigen (HLA) serotypes (DQ2, DR3 and DR7), HIV status, hepatitis C virus (HCV) status and HBV status at the time of listing were obtained.

### 2.2 Outcome measures

The primary study end point was the overall trend in HBV immunity rates among SOT candidates at the time of listing. We also assessed the independent predictors for HBV immunity among our study population of SOT candidates. HBV immunity was defined as an anti‐HBs level ≥ 10 mIU/mL in line with the recommendations of the Advisory Committee on Immunization Practices.^6^

### 2.3 Laboratory testing

Serological markers HBsAg, anti‐HBc, anti‐HBs, HCV antibody (Ab) and HIV antigen/antibody (Ag/Ab) were determined with the Abbott ARCHITECT. HLA serotyping was conducted with Becton Dickinson FACSCanto or FACSLyric.

### 2.4 Statistical analysis

According to the European Association for the Study of the Liver, primary diseases leading to liver transplantation were classified in decompensated cirrhosis, liver cancer, cholestatic diseases, acute hepatic failure, metabolic diseases and other.^30^ Considering the expected lower immune response among patients with decompensated liver cirrhosis, a selected analysis was performed among liver transplant candidates with decompensated cirrhosis versus those without.^31^ In view of the limited literature on HBV immunity by diagnosis in renal, cardiac and pulmonary transplant patients, no subgroup analyses were made in those transplant categories. We stratified HBV immunity rates into two time periods 1997‐2008 and 2009‐2019 to balance the time covered. To determine the significance of universal HBV vaccination on HBV immunity, we stratified SOT candidates into two birth cohorts (born in 1986 or after and birth cohort < 1986). The cut‐off was chosen since children born in 1986 or after were covered by the universal infant HBV vaccination programme in Belgium.^28^, ^29^ Age was grouped as 18‐ to 24‐year‐olds, 25‐ to 40‐year‐olds, 40‐ to 64‐year‐olds and ≥65‐year‐olds as defined by the Medical Subject Headings (MeSH). Selected analysis was performed comparing <40‐year‐olds and ≥40‐year‐olds in line with prior hepatitis B vaccine efficacy studies.^10^, ^32^

Categorical data were analysed using chi‐squared test or Fisher's exact test. Comparison of two and three continuous variables was done with the Mann‐Whitney *U* test or Kruskal‐Wallis *H* test, respectively. Results were expressed either as frequencies (%) or median (interquartile range, IQR). The following predictors for HBV immunity were included in the multivariable logistic regression model: year on waiting list, birth cohort (born in or after 1986 vs birth cohort < 1986), sex (male vs female), obesity (yes vs no), HIV Ag/Ab (positive vs negative) and HCV Ab (positive vs negative). IBM SPSS Statistics for Windows, version 25 (IBM Corp, Armonk, NY), was used for all analyses. The level of statistical significance was set at *P* < .050.

## 3 RESULTS

**Figure 1:**
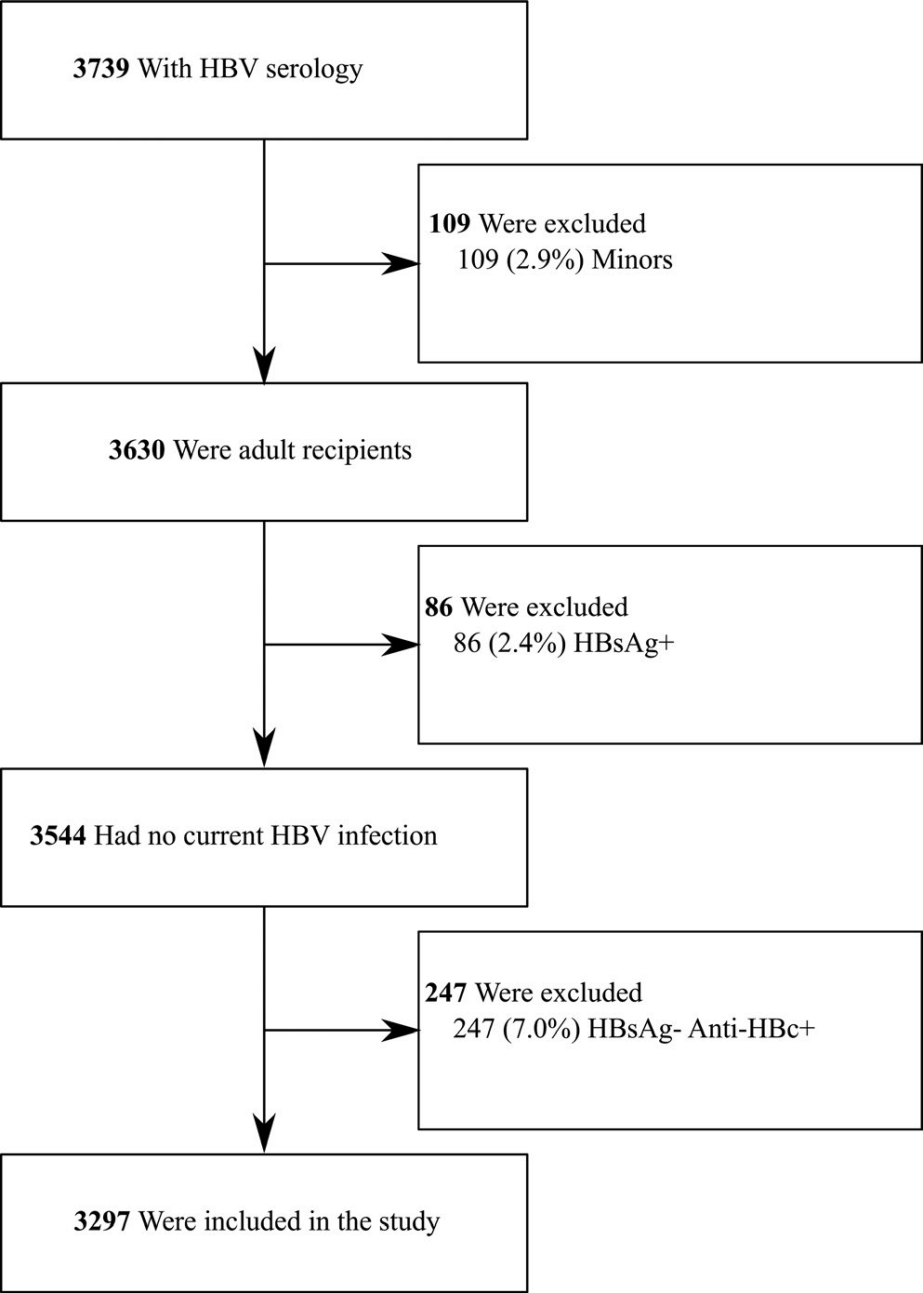
Flow chart of the study. HBV, hepatitis B virus; HBsAg, hepatitis B surface antigen; +, positive, −, negative; Anti‐HBc, hepatitis B core antibodies

Between 1 January 1997 and 21 June 2019, 3739 unique patients with known HBV serology were listed for SOT at our centre. According to inclusion and exclusion criteria, a total of 3297 SOT candidates were included in the current study (Figure 1). Out of 3297 SOT candidates, 1908 (57.9%) had information on race with 1885/1908 (98.8%) being Caucasian. When information on HLA serotype was available, the distribution was as follows: 437/1127 (38.8%) HLA DQ2 positive, 204/1119 (18.2%) HLA DR3 positive and 222/1119 (19.8%) HLA DR7 positive.

### 3.1 Hepatitis B virus immunity by organ and transplant diagnosis

Table 1 illustrates the baseline characteristics among total, liver, renal, cardiac and pulmonary transplantation patients. The prevalence of HBV immunity at the time of listing was 28.5% (940/3297) in the total group, with differences between liver, renal, cardiac and pulmonary transplant candidates: 21.5% (257/1197), 55.3% (483/874), 15.4% (64/415) and 16.8% (136/811), respectively, *P* < .001.

**Table 1:** Baseline characteristics among liver, renal, cardiac and pulmonary transplant candidates (n = 3297)

Characteristics	Total (n = 3297)	Liver transplant (n = 1197)	Renal transplant (n = 874)	Cardiac transplant (n = 415)	Pulmonary transplant (n = 811)	*P* value
Age, years, median (IQR)	57 (15.0)	59 (16.0)	55 (14.0)	54 (17.0)	57 (14.0)	<.001
Birth cohort ≥ 1986^a^, n (%)	168/3297 (5.1)	38/1197 (3.2)	41/874 (4.7)	18/415 (4.3)	71/811 (8.8)	<.001
Male sex, n (%)	1970/3290 (59.9)	717/1191 (60.2)	524/874 (60.0)	325/414 (78.5)	404/811 (49.8)	<.001
BMI, median ± IQR	24 (6.0)	25 (7.0)	23 (7.0)	25 (5.0)	22 (7.0)	<.001
Obesity^b^, n (%)	389/2656 (14.6)	253/1193 (21.2)	39/256 (15.2)	49/401 (12.2)	48/806 (6.0)	<.001
HIV Ab/Ag positive, n (%)	7/3070 (0.2)	2/1100 (0.2)	2/820 (0.2)	0/415 (0.0)	3/735 (0.4)	.569
HCV Ab positive, n (%)	126/3071 (4.1)	97/1158 (8.4)	16/752 (2.1)	3/392 (0.8)	10/769 (1.3)	<.001

Abbreviations: Ab, antibody; Ag, antigen; BMI, body mass index; HBV, hepatitis B virus; HCV, hepatitis C virus; IQR, interquartile range.

^a^ Patients born in 1986 or later are covered by the universal infant HBV vaccination programme in Belgium with catch‐up in 10‐ to 13‐y‐olds.

^b^ Obesity was specified as body mass index > 30 kg/m^2^.

Among 1197 liver transplant candidates, 587 (49.0%) had decompensated cirrhosis. Diagnosis in the remaining 610 patients was as follows: 111 (18.2%) liver cancer, 133 (21.8%) cholestatic diseases, 46 (7.5%) acute/subacute hepatic failure, 95 (15.6%) metabolic diseases and 225 (36.9%) other (Budd‐Chiari: 13; benign liver tumours or polycystic diseases: 97; other liver diseases: 115). HBV immunity rate was 14.8% (87/587) in decompensated cirrhotic patients and 27.9% (170/610) in those without decompensated cirrhosis (*P* < .001).

### 3.2 Hepatitis B virus immunity: overall trend and immunity per birth cohort

**Figure 2:**
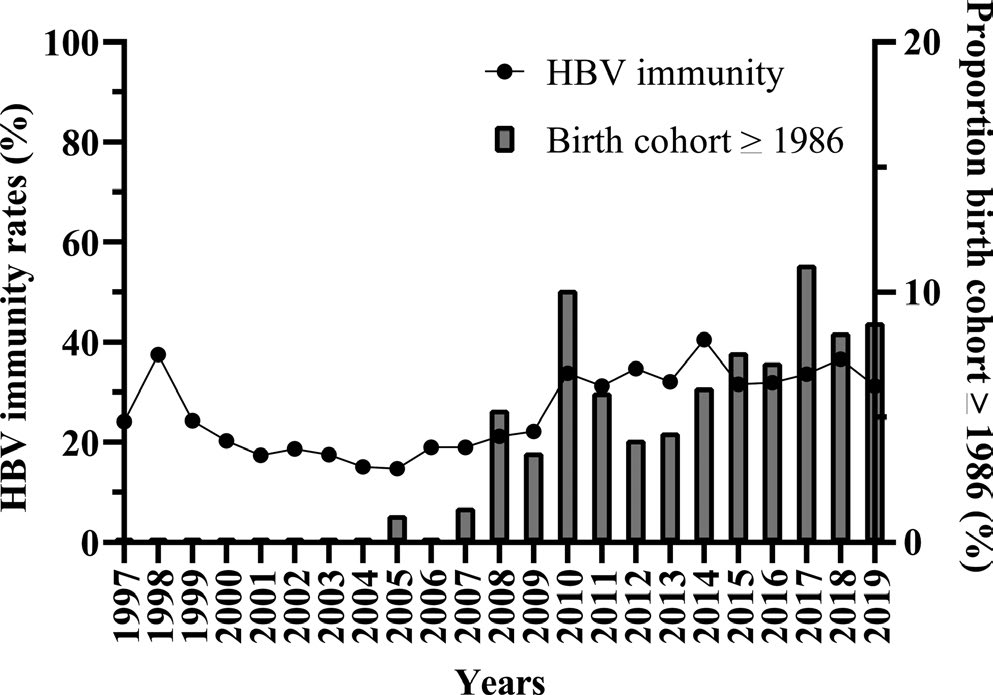
Hepatitis B virus immunity rates, 1997‐2019 (n = 3297) and proportion of patients born in 1986 or later. Abbreviation: HBV: hepatitis B virus. †Immunity was defined as hepatitis B surface antibody levels > 10 mIU/mL. ‡Patients born in 1986 or later are covered by the universal infant HBV vaccination programme in Belgium with catch‐up in 10‐ to 13‐y‐olds

Hepatitis B virus immunity rate was 19.3% (201/1044) in 1997‐2008 and 32.8% (739/2253) in 2009‐2019, *P* < .001 (Figure 2). The proportion of transplant patients born in or after 1986 over the period 1997‐2019 is also presented in Figure 2. These numbers were 1.1% (11/1044) and 7.0% (157/2253) for the period 1997‐2008 and 2009‐2019, respectively, *P* < .001. Compared to the birth cohort ≥ 1986, HBV immunity rates were lower among birth cohort < 1986 (27.8% (869/3122) vs 41.7% (70/168), *P* < .001).

### 3.3 Hepatitis B virus immunity: age and sex

The HBV immunity rates for age groups 18‐ to 24‐year‐olds (young adults), 25‐ to 40‐year‐olds, 40‐ to 64‐year‐olds (middle aged) and ≥65‐year‐olds (aged) were 48.1% (63/131), 36.1% (130/360), 26.6% (619/2324) and 26.6% (128/482), respectively, *P* < .001. Selected analysis was performed comparing < 40‐year‐olds and ≥40‐year‐olds: HBV immunity was seen in 40.7% (186/457) vs 26.5% (754/2840), respectively, *P* < .001.

**Figure 3:**
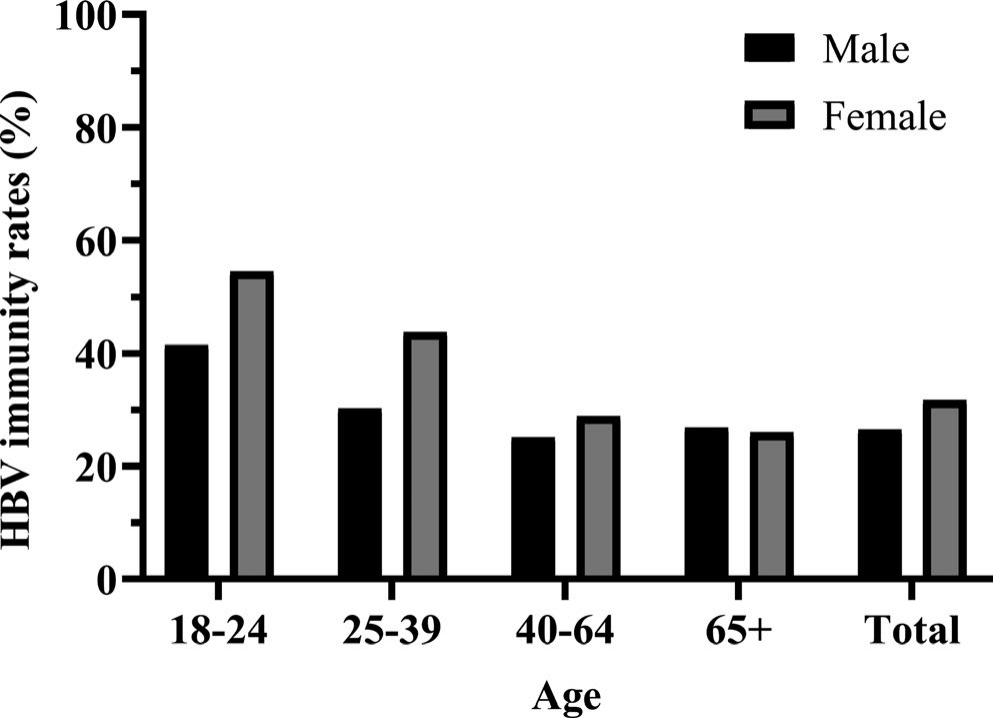
Hepatitis B virus immunity rates by age group and sex (n = 3297). Abbreviation: HBV, hepatitis B virus. †Immunity was defined as hepatitis B surface antibody levels > 10 mIU/mL

The rates for males and females were 26.5% (1448/1970) and 31.7% (902/1320), *P* = .001. HBV immunity rates were highest among 18‐ to 24‐year‐old women (Figure 3). This age group had the highest rate difference between males and females (41.5% (27/65) vs 54.5% (36/66), respectively).

### 3.4 Factors Associated with hepatitis B virus immunity

Table 2 shows the odds ratios (95% confidence interval) for HBV immunity by different risk factors. Male sex was significantly associated with lower immunity rates in multivariable analysis (*P* < .001). Increasing year on waiting list (*P* < .001), younger age (*P* < .001) and birth cohort ≥ 1986 (*P* < .001) indicated a higher rate of HBV immunity.

**Table 2:** Factors predictive of hepatitis B virus immunity (n = 3297)

Variables	Univariate analysis	*P* value	Multivariable analysis	*P* value
	OR (95% CI)		OR (95% CI)	
Year on waiting list	1.04‐1.07	<0.001	1.03‐1.06	<.001
Age	0.97‐0.99	<0.001	0.97‐0.98	<.001
Birth cohort ≥ 1986^b^	1.35‐2.54	<0.001	1.18‐2.66	.006
Male sex	0.67‐0.91	0.001	0.57‐0.85	<.001
Obesity^c^	0.68‐1.16	0.377	0.77‐1.37	.843
HIV Ab/Ag positive	0.19‐5.16	1.000	0.35‐11.02	.448
HCV Ab positive	0.34‐0.86	0.009	0.29‐1.01	.052

Abbreviations: Ab, antibody; Ag, antigen; CI, confidence interval; HCV, hepatitis C virus; OR, odds ratio.

^a^ Immunity was defined as hepatitis B surface antibody levels >10 mIU/mL.

^b^ Patients born in 1986 or later are covered by the universal infant HBV vaccination programme in Belgium with catch‐up in 10‐ to 13‐y‐olds.

^c^ Obesity was specified as body mass index > 30 kg/m^2^.

## 4 DISCUSSION

In our study, only 29% of SOT candidates were HBV immune at the time of listing, leaving 71% at risk of acquiring HBV during or after transplantation and subsequently the risk of developing chronic hepatitis or even fibrotic cholestatic hepatitis. This is of particular importance in case of liver transplantation when anti‐HBc–positive liver donors are used. In such a situation, HBV immunity in the acceptor remains essential to lower the risk of de novo HBV infection in recipients of anti‐HBc–positive donors.^33^, ^34^ Without the use of antiviral prophylaxis, the risk of de novo HBV infection is previously estimated at 47.8% over 35 months in hepatitis B naïve individuals and 9.7% over 40 months in individuals with vaccine immunity.^34^ For non–liver organs, the risk of de novo HBV infection from HBsAg‐negative/anti‐HBc–positive donors is negligible in recipients with HBV immunity, but the risk is up to 5% in those without HBV immunity.^33^, ^35^ The growing organ shortage favours the use of grafts from HBsAg‐negative/anti‐HBc–positive donors as it is estimated that approximately 2 billion people have been exposed to HBV, and excluding all these donors would significantly reduce the global number of available grafts.^36^

An important finding of our study was the increasing HBV immunity rate from 19% in 1997‐2008 to 33% in 2009‐2019. This increase could be explained by the effect of universal vaccination at a young age and in the absence of underlying disease.^6^, ^10^, ^27^, ^28^

Considering the rigorous immunization programme in dialysis patients, the highest HBV immunity rate was observed in renal transplant candidates. Nevertheless, the response rate was still suboptimal in this condition. The management of HBV infection within the haemodialysis unit focuses on identifying the virology status of all patients starting haemodialysis, offering a hepatitis B immunization schedule if indicated and monitoring anti‐HBs levels with repeat immunization in those without protective antibody levels.^37^ However, a concern is that seroprotection rates in these SOT candidates are still suboptimal even with the double‐dose (40 µg) hepatitis B immunization schedule.^12^, ^13^, ^14^, ^15^, ^16^, ^17^, ^18^, ^19^, ^20^, ^21^, ^22^, ^23^, ^24^, ^25^ The expectation of low effectiveness of currently licensed HBV vaccines in renal transplant recipients was shown to be the most important barrier to guideline adherence among Dutch nephrologists.^38^

Similarly for liver transplant candidates, HBV immunity rate was significantly lower in patients with more advanced liver disease: 14.8% in decompensated cirrhotic patients and 27.9% in those without advanced cirrhosis. This again points out that patients with chronic diseases should be systematically vaccinated in the early phase of their disease. As such, the Advisory Committee on Immunization Practices (United States) recommends that all patients at risk for HBV infection (eg chronic liver disease, diabetes mellitus) should be immunized with the hepatitis B vaccine.^6^ However, HBV vaccine coverage is estimated at only 29% in adults with chronic liver disease and 17% in adults with diagnosed diabetes mellitus.^39^, ^40^ In this regard, we currently call for an increased awareness about hepatitis B immunization practices among physicians involved in the management of patients at risk for HBV infection.^6^

Taken together, the low HBV immunity rate (29%) at the time of listing in our study and the expected poor response with the currently available HBV vaccines underline the importance of studies assessing more immunogenic vaccines in SOT candidates. One way to improve the immunogenicity of the current HBV vaccines is to enhance the adjuvant.^41^, ^42^, ^43^, ^44^

This study had some limitations. First, information on hepatitis B vaccination history was not available. Thus, we cannot determine whether low hepatitis B vaccination coverage or suboptimal immune response to hepatitis B immunization accounted for the observed low HBV immunity. Second, SOT candidates in our study were mainly of Caucasian descent and inferences from this study should be drawn with caution for other populations, such as Asians.

In conclusion, this study highlights the low HBV immunity at the time of listing for transplantation even in the candidates for kidney transplantation who received a stringent vaccination schedule before listing. Although an increase in HBV immunity was observed over a 20‐year period, related to the introduction of universal HBV vaccination, opportunities exist to improve HBV vaccine immunity. All patients with a chronic disease, who might be future candidates for organ transplantation, should be vaccinated in an early stage of their disease. This could lead to higher HBV immunity rates and better outcomes in this vulnerable population.

## CONFLICTS OF INTEREST

ÖK received travel grants from Gilead Sciences and his institution received grants from Gilead Sciences, AbbVie, MSD and CyTuVax BV DK received research grants from Astellas, Roche and Novartis, and travel grants from Astellas, CSL Behring and Sandoz. GR has received research grants from AbbVie, MSD and Janssen Pharmaceuticals, and has acted as a consultant/advisor for AbbVie, MSD, Gilead Sciences and Bristol‐Myers Squibb. AL received honorarium for lectures from GSK and Janssen‐Cilag, and all payments were invoiced by the Department of Medical Microbiology, Maastricht UMC+. JV received travel grants from AbbVie, Gilead Sciences and Johnson & Johnson. The following authors reported that they have no conflicts of interest: LD, RV, JVK, JVC, MK, GV and FN.

## AUTHOR CONTRIBUTIONS

ÖK, DK, LD, RV, JVK, JVC, GR, AL, MK, GV, JV and FN contributed to the conception and design of the study. FN supervised ÖK to collect data. Following statistical analysis of data, ÖK drafted the first version of the paper, and the co‐authors critically revised the article and approved the final version to be submitted, including the authorship list.

## ETHICAL APPROVAL

Following Belgian regulation, ethical approval was waived because of the retrospective character of our study.
